# Predictors of Long-Term Mortality in Medically Treated Patients With Chronic Heart Failure in Kosovo

**DOI:** 10.31083/RCM38127

**Published:** 2025-07-30

**Authors:** Gani Bajraktari, Shpend Elezi, Pranvera Ibrahimi, Genc Abdyli, Artan Bajraktari, Arlind Batalli, Afrim Poniku, Frank L. Dini, Michael Y. Henein

**Affiliations:** ^1^Clinic of Cardiology, University Clinical Centre of Kosovo, 10000 Prishtina, Kosovo; ^2^Medical Faculty, University of Prishtina, 10000 Prishtina, Kosovo; ^3^Department of Public Health and Clinical Medicine, Umeå University, SE-901 87 Umeå, Sweden; ^4^Instituto Auxologico IRCCS, 20145 Milan, Italy; ^5^National Heart and Lung Institute, Imperial College London, SW3 6LY London, UK

**Keywords:** heart failure, predictors, echocardiography, outcome, mortality

## Abstract

**Background:**

Heart failure (HF) is a complex clinical syndrome that is associated with high morbidity and mortality. The prognosis of chronic HF in Kosovo has never been objectively assessed and compared with other countries. Thus, this study aimed to investigate the long-term prognostic value of clinical and cardiac function parameters in predicting the mortality of patients in Kosovo with chronic HF.

**Methods:**

This study included 203 consecutive patients with chronic HF who were followed up for a mean of 86 ± 40 months. The primary outcome of the study was all-cause mortality.

**Results:**

During the follow-up period, there were 94 deaths (46.3%). Deceased patients were older (*p* < 0.001), commonly in New York Heart Association (NYHA) class ≥III (*p* < 0.001), had lower 6-minute walk distances (*p* = 0.014), higher prevalence of type 2 diabetes mellitus (T2DM) (*p* = 0.018), raised creatinine (*p* = 0.001), and lower hemoglobin (*p* = 0.004). Moreover, these patients often had left bundle branch block (*p* = 0.001), lower left ventricular (LV) ejection fraction (EF) (*p* < 0.001), larger left atrium (LA) (*p* < 0.001), lower lateral and septal mitral annular plane systolic excursion (MAPSE) values (*p* = 0.001 and *p* < 0.001, respectively), and tricuspid annular plane systolic excursion (TAPSE) (*p* = 0.009), reduced lateral systolic myocardial velocity (s’) (*p* = 0.018), early diastolic myocardial velocity (e’) (*p* = 0.011) and late diastolic myocardial velocity (a’) (*p* = 0.010) velocities, reduced septal e’ (*p* < 0.001) and a’ (*p* = 0.032) velocities, and had higher E/e’ (*p* = 0.021), compared to survivors. Multivariate analysis identified NYHA class ≥III (odds ratio (OR) = 5.573, 95% CI 1.688–18.39; *p* = 0.005), raised creatinine (OR = 1.027, 95% CI 1.006–1.047; *p* = 0.011), advanced age (OR = 1.069, 95% CI 1.011–1.132; *p* = 0.020), enlarged LA (OR = 3.279, 95% CI 1.033–10.41; *p* = 0.044), and left ventricular ejection fraction (LVEF) ≤45% (OR = 3.887, 95% CI 1.221–12.38; *p* = 0.022), as independent predictors of mortality.

**Conclusions:**

In medically treated patients with chronic HF from Kosovo, worse functional NYHA class, impaired kidney function, age, compromised LV systolic function, and enlarged LA were independently associated with increased risk of long-term all-cause mortality.

## 1. Introduction

Despite many recent advances in the diagnosis and treatment of heart failure (HF), this condition remains a complex, heterogeneous, life-threatening clinical syndrome, which is accompanied by high morbidity and mortality, poor quality of life, and high economic burden [1, 2, 3]. HF affects more than 64 million people worldwide; thus, this syndrome is considered a global pandemic [4]. Moreover, the prevalence of HF is predicted to continue rising due to population longevity, which is impacted by advances in medical treatment [5]. Although survival rates of chronic HF patients have already increased over the past decades, the 5-year survival remains close to 50%, and the 10-year survival is less than 35% [6]. HF may be caused by several underlying etiologies, and is often accompanied by cardiac and non-cardiac comorbidities, associated with different adverse outcomes [1]. Previous studies have identified different clinical and echocardiographic predictors of short- and long-term outcomes of HF patients [7, 8, 9, 10, 11, 12]. However, the results are controversial and depend on the type of HF, the age of the patients, the geographic area of residence, and the national economic status. The prognosis of chronic HF in Kosovo has never been objectively assessed and compared with other countries. Therefore, this prospective study aimed to investigate the long-term prognosis of a group of patients admitted with HF at the Clinic of Cardiology, University Clinical Centre of Kosovo. The data for each patient included epidemiological, clinical, and echocardiographic heart structure and function parameters.

## 2. Methods

### 2.1 Study Population

We enrolled 268 patients, who were admitted at the Clinic of Cardiology (Second Division), University Clinical Centre of Kosovo, between September 2009 and November 2013, with the diagnosis of chronic HF, based on the available definitions at the time. All included patients were in New York Heart Association (NYHA) functional classes I–III, including those with ischemic and non-ischemic etiologies [13]. All patients were also receiving conventional, optimized HF treatment, based on the available clinical guidelines in use at the time of the study. During the follow-up period, 65 patients were inaccessible and were therefore excluded from the analysis. The exclusion criteria were, cardiac decompensation (NYHA class IV, those with peripheral edema), recent acute coronary syndrome, severe mitral regurgitation, stroke, limited physical activity due to cardiac and/or non-cardiac causes, significant anemia, more than mild renal failure (raised creatinine >110 mmol/L and estimated glomerular filtration rate (e-GFR) <60 mL/min/1.73 m^2^), and significant chronic obstructive pulmonary disease. A total of 203 patients (mean age 61 ± 10 years, 58.6% female) formed the study population, who were followed up for 86 ± 40 months. Of these included patients, 47% had ischemic etiology (60 of 95 patients were previously revascularized), 40% had hypertensive heart disease, and the remaining 13% had unknown etiologies. Additionally, 17% of the study population were in atrial fibrillation. The patient population comprised 92% who received angiotensin-converting enzyme (ACE) inhibitors or angiotensin II receptor blockers (ARBs), 76% who used beta-blockers, 62% who administered spironolactone, 46% who used diuretics, 6% who used calcium channel blockers, and 4% who used digoxin. This study was approved by the Ethics Committee of the Medical Faculty, University of Prishtina, with reference number 1056/2009. All included patients provided signed informed consent to participate in the study.

The patients were categorized into two groups: HF with preserved EF (HFpEF: EF ≥45%) and HF with reduced EF (HFrEF: EF <45%) based on the recorded mean left ventricular (LV) ejection fraction (EF) during the hospital stay [14, 15].

### 2.2 Data Collection

The medical history of each patient was obtained, and a clinical examination was undertaken in all patients at the time of enrollment. Biochemical tests, including lipid profile, blood glucose level, kidney function tests, and hemoglobin, were also performed in all patients. Body mass index (BMI) was measured using weight and height measurements, and the body surface area (BSA) was calculated using the Du Bois formula: BSA (m^2^) = 0.007184 × (height in cm)^0.725^ × (weight in kg)^0.425^ [16]. The waist/hips ratio was calculated from the waist and hips measurements in all patients.

### 2.3 Cardiac Structure and Function 

Cardiac structure and function were studied using conventional Doppler echocardiography. All echocardiographic examinations were performed by a single operator using the Philips Intelligent E-33 system (Philips Healthcare, Andover, MA, USA), equipped with a multi-frequency transducer and harmonic imaging as needed. Images were obtained during quiet expiration, while the patient was in the left lateral decubitus position. LV end-diastolic and end-systolic dimensions, as well as interventricular septal and posterior wall thickness, were measured based on the recommendations of the European and American Society of Echocardiography [17, 18]. Left ventricular volumes and EF were calculated using the modified Simpson’s method. The M-mode technique was used to study left and right ventricular (RV) long-axis function, placing the cursor at the lateral and septal angles of the mitral annulus and the lateral angle of the tricuspid annulus [19]. Long axis measurements were identified as lateral and septal mitral annular plane systolic excursion (MAPSE) and tricuspid annular plane systolic excursion (TAPSE). LV and RV long-axis myocardial velocities were also studied using the Doppler myocardial imaging technique (tissue Doppler imaging, TDI), from the apical 4-chamber view. Longitudinal velocities were obtained using the pulsed wave Doppler sample volume placed at the basal part of the LV lateral and septal segments, as well as the basal part of the RV free wall. Systolic (s’) as well as early and late (e’ and a’) diastolic myocardial velocities were measured with the gain optimally adjusted. The mean value of the lateral and septal LV velocities was calculated [20]. Left atrial (LA) size was estimated in the parasternal long-axis view from the trailing edge of the posterior aortic wall to the leading edge of the posterior LA wall, in systole. The LA cavity was described as enlarged if the transverse diameter was ≥47 mm for women and ≥52 mm for men [21]. Diastolic LV function was assessed from LV filling velocities using the spectral Doppler technique with the pulsed wave Doppler sample volume placed at the tips of the mitral valve orifice during a brief apnea. Peak LV early (E wave) and late (A wave) LV filling velocities were measured, and the E/A ratio was calculated. Trans-mitral E wave deceleration time (DT) was also measured from peak E wave to the end of its deceleration. The E/e’ ratio was calculated from the trans-mitral E wave and mean lateral and septal segments myocardial e’ wave velocities, to reflect raised LA pressure. LV filling pattern was considered “restrictive” when the E/A ratio was >2.0, the E wave deceleration time was <140 ms, and the LA dilated with a transverse diameter was >40 mm [22]. Total LV filling time was measured from the onset of the E wave to the end of the A wave, and ejection time from the onset to the end of the aortic Doppler pulsed wave flow velocity. The total isovolumic time and Tei index were calculated from the two measurements to reflect LV dyssynchrony. Mitral regurgitation severity was graded as mild, moderate, or severe based on the relative jet area to that of LA, as well as the flow velocity profile. This assessment was performed in accordance with the recommendations of the European and American Society of Echocardiography [23, 24]. Color Doppler and continuous-wave Doppler were used for the assessment of tricuspid regurgitation. A pressure drop at retrograde trans-tricuspid >35 mmHg was considered as pulmonary hypertension [25]. The Doppler and M-mode recordings were registered at a fast speed of 100 mm/s with a superimposed electrocardiography (ECG) (lead II).

### 2.4 Six-Minute Walk Test

A 6-minute walk test (6-MWT) was performed within 24 hours of the echocardiographic examination. The test was performed in a level hallway corridor and administered by a specialized nurse, who was blinded to the echocardiographic findings. All study patients, who were on regular medical treatment, were informed of the purpose and protocol of the 6-MWT [26, 27, 28]. Patients were instructed to walk as far as possible for 6 minutes, turning 180° at the end of the corridor. Patients were not influenced by walking speed and walked unaccompanied. The supervising nurse measured the total distance patients walked at the end of the 6 minutes.

### 2.5 Follow-Up 

After the baseline Doppler echocardiogram, all study patients were followed for a mean period of 86 ± 40 months. The study outcome endpoint was all-cause mortality. Follow-up data were obtained through regular visits, telephone calls, or hospital records.

### 2.6 Statistical Analysis

Data are presented as the mean ± standard deviation (SD) or proportions (% of patients). Continuous data were compared using a two-tailed unpaired Student *t*-test, and discrete data were compared using a chi-square test. Predictors of mortality were identified through univariate and multivariate logistic regression analyses, employing the stepwise selection method. Univariate logistic regression was used to identify potential predictors of all-cause mortality. Variables with *p* < 0.10 in univariate analysis were considered for inclusion in the multivariate model. The multivariate analysis was performed using binary logistic regression with a backward stepwise elimination method, based on likelihood ratio statistics, to determine independent predictors of mortality. Odds ratios (ORs) with 95% confidence intervals (CIs) were reported. A *p*-value < 0.05 (two-tailed) was considered statistically significant. The variables were dichotomized according to the following threshold values: EF ≤45% and LA diameter ≥47 mm for women and ≥52 mm for men. Kaplan–Meier curves were constructed, and log-rank tests were used to test for differences between survival curves. A Venn diagram was used to identify the accuracy of parameter combinations in predicting mortality.

## 3. Results

### 3.1 Clinical Data, Survivors Versus Non-Survivors

A total of 94/203 (46.3%) patients had died at the end of the follow-up period of 86 ± 40 months. The deceased were older (*p* < 0.001), with a higher prevalence of type 2 diabetes mellitus (T2DM) (*p* = 0.018), NYHA class ≥III (*p* < 0.001), left bundle branch block (*p* = 0.001), lower 6-minute walk distance (*p* = 0.014), higher creatinine (*p* = 0.001), and lower hemoglobin (*p* = 0.004) than survivors (Table 1).

**Table 1.** **Clinical data for both endpoint patient groups with chronic heart failure**.

Variable	All study patients (n = 203)	Survivors (n = 109)	Deceased (n = 94)	*p*-value
Age (years)	61 ± 10	58 ± 10	65 ± 10	<0.001
Female (n, %)	119 (58.6)	70 (64.2)	49 (52.1)	0.088
Smoking (n, %)	55 (27.8)	26 (24.3)	29 (30.9)	0.343
Diabetes mellitus (n, %)	56 (28.1)	22 (20.8)	34 (36.6)	0.018
Arterial hypertension (n, %)	154 (77.4)	88 (83)	66 (71)	0.061
Atrial fibrillation (n, %)	34 (16.7)	16 (14.7)	18 (19.1)	0.541
HFrEF (n, %)	65 (32)	16 (14.7)	49 (52.1)	<0.001
NYHA Class ≥III (n, %)	57 (28.1)	10 (9.2)	47 (50)	<0.001
LBBB (n, %)	35 (17.2)	10 (9.2)	25 (26.6)	0.001
Waist/hips ratio	0.96 ± 0.07	0.94 ± 0.07	0.97 ± 0.08	0.008
BMI (kg/m^2^)	29 ± 4	29 ± 4	28 ± 4	0.054
Fasting glucose (mmol/L)	6.8 ± 3.0	6.7 ± 2.8	6.9 ± 3.1	0.636
Total cholesterol (mmol/L)	4.7 ± 1.3	4.87 ± 1.3	4.49 ± 1.2	0.090
Triglycerides (mmol/L)	1.6 ± 0.7	1.68 ± 0.8	1.54 ± 0.7	0.298
Blood urea nitrogen (mmol/L)	9.3 ± 5.9	6.7 ± 2.6	11.4 ± 6.9	<0.001
Creatinine (µmol/L)	101 ± 52	85 ± 25	114 ± 64	0.001
Hemoglobin (g/dL)	12.6 ± 2.2	13 ± 2.1	12.2 ± 2.3	0.004
6-MWT distance (m)	283 ± 114	308 ± 108	253 ± 115	0.014
Baseline HR (beats/min)	77 ± 13	75 ± 13	78 ± 14	0.118

NYHA, New York Heart Association; LBBB, left bundle branch block; 6-MWT, six-minute walk test; BMI, body mass index; HR, heart rate; HFrEF, heart failure with reduced ejection fraction.

### 3.2 Cardiac Function, Survivors Versus Non-Survivors

Non-survivors had lower left ventricular ejection fraction (LVEF) (*p* < 0.001), larger LA (*p* < 0.001), reduced lateral and septal MAPSE (*p* = 0.001 and *p* < 0.001, respectively), as well as TAPSE (*p* = 0.009); moreover, the non-surviving patients had reduced lateral s’ (*p* = 0.018), e’ (*p* = 0.011) and a’ (*p* = 0.010) velocities, reduced septal e’ (*p* < 0.001) and a’ (*p* = 0.032) velocities, and a higher E/e’ (*p* = 0.021), compared to survivors. Total isovolumic time and Tei index were not different between the two groups (Table 2).

**Table 2.** **Echocardiographic data for both endpoint patient groups with chronic heart failure**.

Variable		All study patients (n = 203)	Survivors (n = 109)	Deceased (n = 94)	*p*-value
Systolic LV function					
	LV ejection fraction (%)	52 ± 16	59 ± 15	49 ± 16	<0.001
	MAPSE lateral (cm)	1.3 ± 0.4	1.3 ± 0.4	1.1 ± 0.3	0.001
	MAPSE septal (cm)	1.0 ± 0.3	1.1 ± 0.3	0.9 ± 0.3	<0.001
	Left atrium diameter (cm)	4.3 ± 0.8	4.1 ± 0.8	4.6 ± 0.7	<0.001
	Lateral s’ wave (cm/s)	5.7 ± 1.8	6.0 ± 1.8	5.4 ± 1.7	0.018
	Septal s’ wave (cm/s)	5.0 ± 1.6	5.15 ± 1.6	4.65 ± 1.7	0.071
Diastolic LV function					
	E/A ratio	1.1 ± 0.9	0.93 ± 0.6	1.3 ± 1.2	0.015
	E wave deceleration time (ms)	179 ± 57	186 ± 51	171 ± 61	0.069
	Lateral e’ (cm/s)	7.1 ± 2.9	7.7 ± 3.2	6.5 ± 2.5	0.011
	Septal e’ (cm/s)	5.6 ± 2.1	6.2 ± 2.1	4.6 ± 1.8	<0.001
	E/e’ (cm/s)	9.9 ± 6.7	8.8 ± 5.9	11 ± 7.3	0.021
	Lateral a’ (cm/s)	8.0 ± 3.1	8.5 ± 2.9	7.3 ± 3.2	0.010
	Septal a’ (cm/s)	8.0 ± 2.4	8.3 ± 3.3	7.2 ± 2.4	0.032
Global LV function					
	T-IVT (s/min)	9.5 ± 5.2	9.6 ± 5.8	9.3 ± 4.1	0.523
	Tei index	0.42 ± 0.3	0.49 ± 0.3	0.56 ± 0.3	0.118
RV function					
	TAPSE (cm)	2.2 ± 0.6	2.3 ± 0.5	2.1 ± 0.6	0.009
	Right e’ (cm/s)	9.6 ± 3.8	9.9 ± 3.8	9.2 ± 3.8	0.244
	Right a’ (cm/s)	12.2 ± 4.6	12.1 ± 4.6	12.5 ± 4.6	0.673
	Right s’ (cm/s)	8.8 ± 3.3	8.8 ± 2.9	8.8 ± 3.9	0.930

LV, left ventricle; RV, right ventricle; A, atrial diastolic velocity; E, early diastolic filling velocity; T-IVT, total isovolumic time; s’, systolic myocardial velocity; e’, early diastolic myocardial velocity; a’, late diastolic myocardial velocity; MAPSE, mitral annular plane systolic excursion; TAPSE, tricuspid annular plane systolic excursion.

### 3.3 Predictors of Mortality in the Whole Patient Population

**Fig. 1.**
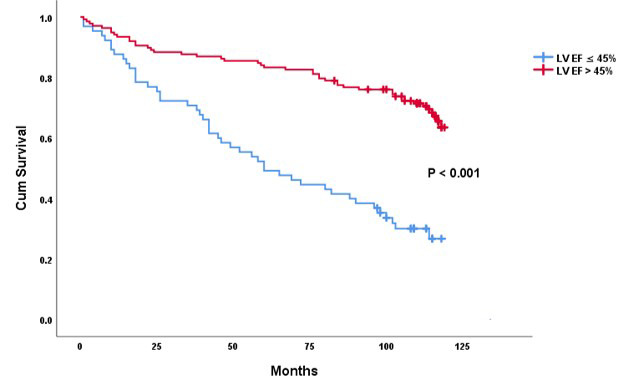
**Survival free of death predicted by LVEF ≤45% vs. LVEF >45%**. LVEF, left ventricular ejection fraction.

In the univariate analysis, age, NYHA class ≥III, enlarged LA, LVEF ≤45%, reduced septal MAPSE (*p* < 0.001 for all), lateral MAPSE (*p* = 0.001) and TAPSE (*p* = 0.011), raised creatinine (*p* = 0.001 for all), in addition to reduced hemoglobin (*p* = 0.005), diabetes melitus (DM) (*p* = 0.012), compromised 6-minute walk distance (*p* = 0.017), raised E/A ratio (*p* = 0.021), reduced lateral s’ (*p* = 0.021) and high E/e’ ratio (*p* = 0.028), were predictors of all-cause mortality. The multivariate analysis identified NYHA class ≥III (OR = 5.573, 95% CI 1.688–18.39; *p* = 0.005), raised creatinine (OR = 1.027, 95% CI 1.006–1.047; *p* = 0.011), advanced age (OR = 1.069, 95% CI 1.011–1.132; *p* = 0.020), enlarged LA (OR = 3.279, 95% CI 1.033–10.41; *p* = 0.044), and LVEF ≤45% (OR = 3.887, 95% CI 1.221–12.38; *p* = 0.022; Fig. 1), as independent predictors of mortality (Table 3).

**Table 3.** **Predictors of mortality during follow-up in patients with heart failure**.

	Univariate analysis			Multivariate analysis		
Variable	OR	95% CI	*p*-value	OR	95% CI	*p*-value
Age	1.082	(1.047–1.119)	<0.001	1.069	(1.011–1.132)	0.020
Female gender	0.607	(0.345–1.065)	0.082			
BMI	0.932	(0.866–1.002)	0.057			
Smoking	1.424	(0.765–2.650)	0.264			
Diabetes	2.241	(1.195–4.204)	0.012	1.716	(0.536–5.487)	0.363
Cholesterol	0.789	(0.597–1.041)	0.940			
Creatinine	1.024	(1.010–1.038)	0.001	1.027	(1.006–1.047)	0.011
Hemoglobin	0.774	(0.647–0.925)	0.005	1.021	(0.758–1.376)	0.889
NYHA class ≥III	9.900	(4.603–21.29)	˂0.001	5.573	(1.688–18.39)	0.005
Severely enlarged left atrium	5.485	(2.650–11.36)	˂0.001	3.279	(1.033–10.41)	0.044
LVEF ≤45%	6.329	(3.248–12.33)	˂0.001	3.887	(1.221–12.38)	0.022
E/A	1.587	(1.071–2.351)	0.021			
6-minute walk distance	0.996	(0.992–0.999)	0.017			
MAPSE lateral	0.214	(0.085–0.537)	0.001			
MAPSE septal	0.104	(0.033–0.325)	˂0.001			
TAPSE	0.469	(0.263–0.838)	0.011	0.999	(0.411–2.266)	0.936
S’ lateral	0.798	(0.658–0.967)	0.021			
S’ septal	0.807	(0.636–1.023)	0.076			
E/e’	1.060	(1.006–1.117)	0.028			

LVEF, left ventricular ejection fraction; MAPSE, mitral annular plane systolic excursion; TAPSE, tricuspid annular plane systolic excursion; NYHA, New York Heart Association; BMI, body mass index; HR, heart rate; A, atrial diastolic velocity; E, early diastolic filling velocity; e’, early diastolic myocardial velocity.

**Fig. 2.**
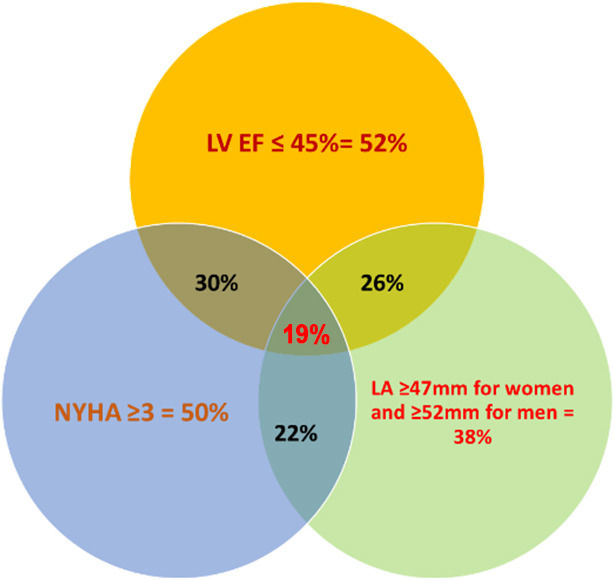
**Venn diagram of the percentages of the deceased patients with HF who had an LVEF ≤45%, were in NYHA class III, and had an enlarged LA, as well as all three abnormalities combined**. HF, heart failure; NYHA, New York Heart Association; LVEF, left ventricular ejection fraction; LA, left atrium.

Predictor accuracies were not significantly different from one another, except for the enlarged LA, which was modestly lower than the others. In total, 52% of the deceased patients had an LVEF ≤45%, while 50% were in NYHA class III, and 38% had an enlarged LA. These combined disturbances were present in 19% of the deceased (Fig. 2).

### 3.4 Predictors of Mortality Based on an LVEF >45% Versus ≤45%

During the follow-up period, 45/138 (32.3%) patients with an EF >45% died, and 49/65 (75.4%) patients with an EF ≤45% died; the difference in the prevalence of death between the two groups was significant (*p* < 0.001). In patients with an LVEF >45%, raised creatinine (OR = 1.025, 95% CI 1.004–1.046; *p* = 0.021) and a NYHA class ≥III (OR = 9.299, 95% CI 1.985–43.56; *p* = 0.005) were independent predictors of mortality. In contrast, the multivariate analysis did not identify any independent predictor of mortality for those with HF and an LVEF ≤45% during follow-up (Table 4).

**Table 4.** **Multivariate predictors of mortality in HF patients with LVEF >45% vs. LVEF ≤45%**.

	LVEF >45%			LVEF ≤45%		
Variable	OR	95% CI	*p*-value	OR	95% CI	*p*-value
Age	1.033	(0.952–1.121)	0.436	1.085	(0.980–1.201)	0.118
Diabetes	2.973	(0.642–13.77)	0.164	1.092	(0.123–9.759)	0.983
Creatinine	1.025	(1.004–1.046)	0.021	1.046	(0.989–1.107)	0.117
Hemoglobin	1.020	(0.710–1.466)	0.913	1.460	(0.676–3.152)	0.355
NYHA class ≥III	9.299	(1.985–43.56)	0.005	6.717	(0.484–93.13)	0.156
Enlarged left atrium	5.145	(0.994–26.64)	0.051	2.973	(0.311–28.46)	0.344
TAPSE	0.600	(0.197–1.852)	0.368	2.424	(0.340–17.42)	0.376

NYHA, New York Heart Association; TAPSE, tricuspid annular plane systolic excursion; LVEF, left ventricular ejection fraction; HF, heart failure.

## 4. Discussion

### 4.1 Findings 

This follow-up study of a cohort of Kosovo patients with chronic HF who were clinically stable on conventional HF medical treatment identified several factors that predicted all-cause mortality within a follow-up period of 86 ± 46 months. Indeed, a NYHA class ≥III, high creatinine, age, an LVEF ≤45%, and an enlarged LA were independent predictors of mortality in these patients. Stratifying the patients according to LVEF measurements >45% and ≤45% resulted in significantly different results. All-cause mortality of patients with an LVEF >45% was independently predicted by high creatinine, NYHA class ≥III, and enlarged LA. Comparatively, all-cause mortality of those patients with an LVEF ≤45% could not be predicted by any of the assessed cardiac or systemic variables. The three predictors of mortality coexisted in only 19% of patients. Finally, all-cause mortality was significantly higher in patients with an LVEF ≤45% compared to those with an LVEF >45%.

### 4.2 Data Interpretation

In this study, the deceased patients were older, predominantly smokers, with worse kidney function, worse NYHA class, and shorter 6-minute walk test distance. These patients also exhibited worse cardiac function, characterized by a lower LVEF, a larger LA, and clear signs of elevated LV filling pressures, yet a similar degree of LV global dyssynchrony, as assessed by total isovolumic time and Tei index. Therefore, although clinically stable at the beginning of the study, the deceased patients were in worse clinical condition compared to the survivors. This probably justifies their higher all-cause mortality, which was likely contributed to by many other clinical factors in addition to cardiac dysfunction. Interestingly, the accuracy of the above predictors was not significantly different, except for that of the enlarged LA, which is secondary to the primary LV systolic and diastolic disturbances. The coexistence of the three predictors was identified in only 19% of the deceased, thus confirming the different hemodynamic statuses of the patients, with some having a worse LVEF and others a stiffer cavity with raised filling pressures and a dilated LA. Splitting patients into two groups based on LVEF (≤45% and >45%) added more clarity to the picture, as despite similar ages between the two groups, the mortality rate of the LVEF ≤45% group was over double that of patients with an LVEF >45%. Moreover, the all-cause mortality for the group with an LVEF >45% could be predicted by a NYHA class >III, raised creatinine, and enlarged LA, consistent with worse cardiac condition as well as renal impairment. These results reflect the nature of the clinical outcome we initially established in this study, but failed to predict the all-cause mortality of the group with an LVEF ≤45%. Although the LVEF was lower in the latter group, the lack of mortality predictors reflects the potential multiple pathologies these patients had over and above HF with LVEF >45%; all of which promoted the significantly higher mortality exhibited in this study. Our findings on the overall all-cause mortality rate in the cohort align with previously published data from different countries [29, 30, 31], despite significant differences in the ratio between patient groups when classified according to LVEF [29, 32, 33, 34]. Moreover, the mortality rate did not differ significantly from that in developed countries [35]. The likely explanation of the lower mortality in the LVEF >45% group is the potential underdiagnosis, whether due to limited referrals to medical centers or missed diagnoses by local general practitioners, as well as patients managed as having hypertension-related symptoms, rather than HF. It is worth noting that predictors of long-term mortality vary across different geographically and economically developed countries and are often influenced by various underlying conditions and treatments [36, 37, 38]. On the other hand, the data from this study are unique in terms of geographical territory, treatment, and mortality prediction.

### 4.3 Study Limitations

This study has some obvious limitations. Although it was a prospective study in its nature, several patients did not fulfil the inclusion criteria and therefore had to be excluded; hence, the study cannot be described as consecutive. Furthermore, the study did not include myocardial deformation measurements, as these were not part of the routine clinical protocol used to assess the patients. The cohort size is modest, so we could not classify the patients into small groups according to the most likely clinical diagnosis that led to death. We also did not use the current HF classification based on LVEF, which was not available at the time of planning. Modern medical treatments, according to current clinical guidelines, were not available at the time; this could have altered our results. The left atrial diameter was used in this study, rather than the more robust indices of the left atrium, which can also assess left atrial function.

### 4.4 Clinical Implications

In a group of patients from Kosovo with chronic HF, age, worse NYHA class, and severity of LV dysfunction, in addition to renal impairment, predicted all-cause mortality. Patients with an LVEF ≤45% presented a worse prognosis than those with an LVEF >45%, suggesting a need for the former group to have critical regular medical reviews with multidisciplinary experts. The significant difference in mortality rate between patient subgroups, according to an LVEF measurement of >45% and ≤45%, warrants specific referral to specialized centers where optimal treatment is offered. Regular assessment of N-terminal prohormone of brain natriuretic peptide (NT-pro-BNP) in all symptomatic patients with a history of long-standing hypertension, irrespective of LVEF, should guarantee optimum patient stratification and management.

## 5. Conclusion 

In medically treated patients from Kosovo with chronic HF, worse functional NYHA class, impaired kidney function, age, compromised LV systolic function, and enlarged LA were independently associated with an increased risk of long-term all-cause mortality, particularly in patients with an LVEF above 45%. The relevance of these predictors in patients with an LVEF ≤45% needs to be assessed in a larger cohort.

## Availability of Data and Materials

The datasets used and analyzed during the current study are available from the corresponding author upon reasonable request.
